# A bidirectional growth mechanism for a stable lithium anode by a platinum nanolayer sputtered on a polypropylene separator†

**DOI:** 10.1039/c8ra02140f

**Published:** 2018-04-09

**Authors:** Kaihua Wen, Lili Liu, Shimou Chen, Suojiang Zhang

**Affiliations:** Beijing Key Laboratory of Ionic Liquids Clean Process, CAS Key Laboratory of Green Process and Engineering, Institute of Process Engineering, Chinese Academy of Sciences Beijing 100190 P. R. China chenshimou@ipe.ac.cn sjzhang@ipe.ac.cn; University of Chinese Academy of Sciences Beijing 100049 P. R. China; Key Laboratory of Cosmetic, China National Light Industry, School of Science, Beijing Technology and Business University Beijing 100048 China

## Abstract

The issue of uncontrollable Li dendrite growth, caused by irregular lithium deposition, restricts the wide applications of Li metal based high energy batteries. In this paper, a polypropylene separator with a sputtered platinum nanolayer has been developed to improve the stability of the Li metal anodes. It was found that cells using the modified separators resulted in a smooth Li surface and a stable “electrode–electrolyte” interface. On the one hand, platinum nanolayers can enhance the mechanical properties and micro-structures of commercial polypropylene separators. On the other hand, platinum nanolayers provide stable Li deposition during repeated charging/discharging by a bidirectional growth mechanism. After long-time cycling, the dendrites from opposite directions and dead Li are integrated into a flat and dense new-formed Li anode, decreasing the risk of low Coulombic efficiency and cycling instability that may end in cell failure. This design may provide new ideas in next-generation energy storage systems for advanced stable metallic battery technologies.

## Abstract 2

Stable Li nucleation and deposition were achieved by applying a platinum-modified-separator with a bidirectional lithium growth mechanism.

## Graphical abstract

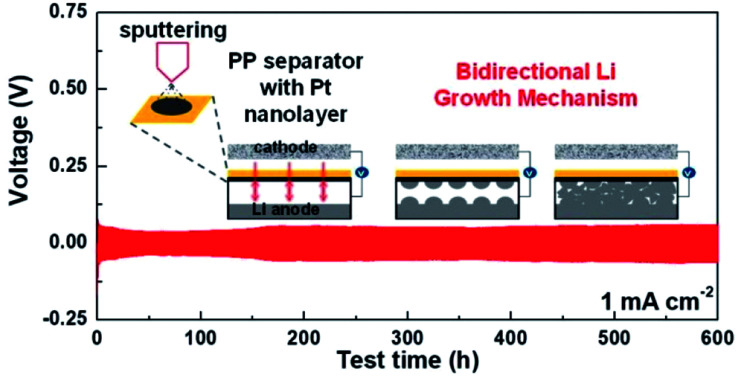

## Introduction

Lithium (Li) metal has been pursued as the most promising anode for high energy electrochemical systems.^1,2^ The tendency of Li to form rough, dendritic deposits over extended charge–discharge cycles is an impediment to widespread deployment of such cells.^3^ During the operation of a Li metal battery, Li-ions transport between a cathode and an anode. Unlike the carbon-based anodes, Li-ions obtain electrons on the Li anodes and then form Li depositions on the Li surface. In the past decades, researchers have carried out mechanistic studies on Li deposition and observed the steps of Li growth by simulations and advanced characterization techniques.^4–6^ Generally, Li-ions firstly form mushroom-/sphere-like roots on the Li surface, and these nuclei give the Li anodes rough surfaces, which leads to inhomogeneous current distributions and high local current densities, especially on the tips of the Li nuclei.^7^ In the stage of Li growth, Li tends to form unstable dendrites on the nuclei. Eventually, the Li dendrites penetrate through the separator leading to short-circuit that may end in cell failure by either voltage or thermal run-away.

To minimize the cause of Li dendrites, researchers have made a lot of efforts for decades. A major approach for stabilizing Li anodes is developing novel electrolyte systems, including solid-state-electrolytes and electrolyte additives.^8–10^ Solid-state-electrolytes, including solid inorganic electrolytes (SIEs) and solid polymer electrolytes (SPEs), possess high mechanical strength to suppress the formation of lithium dendrites.^2^ However, high interfacial resistances between electrolytes and electrodes and low ionic conductivities at room temperature pose the serious barriers for Li-ion transportation in the above two systems. The combination of SIEs and SPEs can enhance the application of solid-state electrolytes, such as a polymer/ceramic/polymer sandwich electrolyte,^11^ Al_2_O_3_–PEO composite electrolyte,^12^ flexible solid ion-conducting membrane by electrospinning,^13^*etc.* In addition, novel additives for liquid electrolytes such as HF,^14^ LiF,^15^ In(TFSI)_3_,^16^ CsPF_6_,^17^ Li_2_S_8_–LiNO_3_ (ref. 18) and ionic liquids^19,20^ can modulate the deposition of Li to form a regular phase. Another approach for smooth Li anode is constructing 3D nanoscale Li anodes and functional collectors. Networks or cages with lithiophilic sites and nano confinement, such as glass fibers,^21^ ZnO-coated polyimide/Li anode,^22^ rGO/Li anode,^23^ kimwipe paper^24^ and N-doped graphene/Li anode,^25^ can make uniform Li flux and stable Li deposition. Additionally, a thin alloy film can be formed on the surface of collectors made of B, Sn, Al, Mg, with some improvement in the reversibility of Li anodes.^26,27^ On the other hand, uniform Li-ions distribution and smooth Li surfaces have been achieved by using a modified separator or inter-protective layer between Li anode and separator/electrolyte. Nanoparticles, like inert ceramics (Al_2_O_3_, TiO_2_, SiO_2_, *etc.*) and 2D materials (such as boron nitride nanotubes) were composited with commercial separators by some coating methods, and modified separators can suppress Li dendrites growth by the developed modulus.^28^ Recently, new methods have been reported to stable Li growth by offering positive Li deposition sites, which is able to change Li dendrites growth to a positive direction. Xie *et al.*^29^ developed a separator with functionalized nanocarbon with immobilized Li ions to form a bidirectional Li growth during cycling and thereby prevent the penetration from the anode site. In another study, Yang and co-workers used a micro-compartmented anode arrays to direct lateral growth of Li growth and then achieve ultimate safe batteries.^30^

**Fig. 1:**
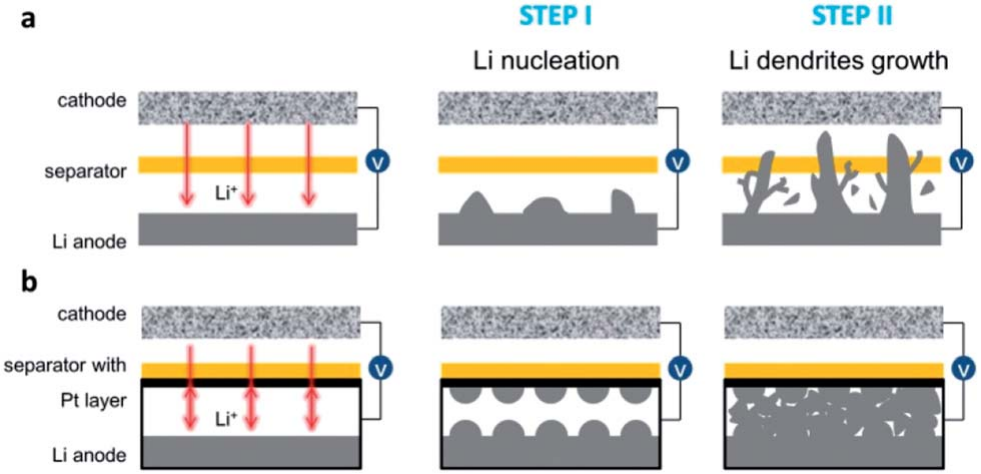
Schematic illustration of the dendrite growth in cells with bare (a) PP and (b) PP@Pt separators.

In this work, a one-step sputter technology on one side of polypropylene separator was reported to make a platinum modified polypropylene (PP) separator, which can achieve reversible Li metal batteries. Previously, it is reported that platinum (Pt) showing solubility in lithium can provide both an electron conductor framework and lithiophilic sites,^31^ which operated differently in the process of Li nucleation from the common Cu current collector.^32,33^Fig. 1 compares the schematic illustration of the dendrite growth in both bare PP and PP@Pt separators. In commercial separators (Fig. 1a), Li dendrites get nucleation on the Li anode side in the initial stage and become large Li-tree during the following long cycling operation, resulting low Coulombic efficiency and eventual penetration to the cathode side.^34^ As shown in Fig. 1b, we hypothesize that the electron conductive Pt nanolayer can provide lithiophilic sites for uniform Li deposition by a bidirectional growth mechanism, resulting Li nucleation and growth from two opposite sides from Li and separator surfaces, respectively. After long-time cycling, the dendrites from opposite directions and dead Li are integrated into a flat and dense new-formed Li anode, decreasing the risk of low Coulombic efficiencies and cycling instability that may end in cell failure.

## Results and discussion

**Fig. 2:**
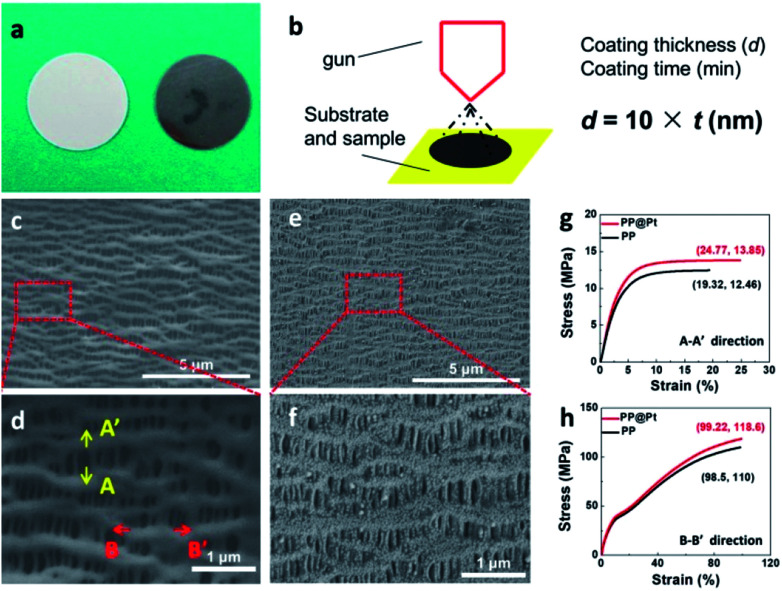
(a) Digital images of PP separators before (left) and after (right) Pt sputtering. (b) Schematic illustration of DC magnetron sputtering and the relationship between coating thickness and coating time. SEM images of (c and d) PP and (e and f) PP@Pt separators. Stress–strain curves of PP and PP@Pt separators in the directions of (g) A–A′ and (h) B–B′.

Polypropylene separator, providing high porosity, high absorption of liquid electrolytes and high flexibility, is widely used in practical application.^35^ To obtain a Pt nanolayer on commercial PP separator, a DC magnetron sputtering method was used in this work. Fig. 2a shows the digital pictures of PP separator before and after sputtering Pt. The thickness of the Pt nanolayer (*d*, nm) is determined by the spraying time (*t*, min) and sputtering current during operation.^36^ In this work, a proportional relationship between *d* and *t* performed as the following equation, *d* = *k* × *t* (nm), where *k* = 10 nm min^−1^ at a DC current of 30 mA (Fig. 2b). Scanning electron microscope (SEM) was applied to present the morphology of PP separators with and without Pt modified layer. As shown in Fig. 2c, the PP separator showed the structure of polymer matrix with regular nano-channels for Li-ions transportation. After sputtering for 12 min, a Pt layer with 120 nm thickness was prepared as a composite separator. Pt nanoparticles were adhered on the matrix of PP separator (Fig. 2e and f). SEM images of PP@Pt separators with thickness of 25 and 250 nm were performed in Fig. S1.† PP separator with 2.5 nm Pt layer is too thin to be distinguished, while 250 nm Pt layer is too thick to block a large amount of nanochannels for Li-ions transportation. Thus, the thickness of 120 nm was selected to be investigated in this work. The magnified image shown in Fig. 2d distinguished the microstructure in longitude (A–A′) and latitude (B–B′) of PP separator produced by a uniaxial tensile technology, resulting different stress–strain performances in the two directions.^37^ The stress–strain curves for separators are presented in Fig. 2g and h. In both A–A′ and B–B′directions, PP@Pt separator shows a slightly enhancement of the modulus by Pt modification than bare PP separator.

**Fig. 3:**
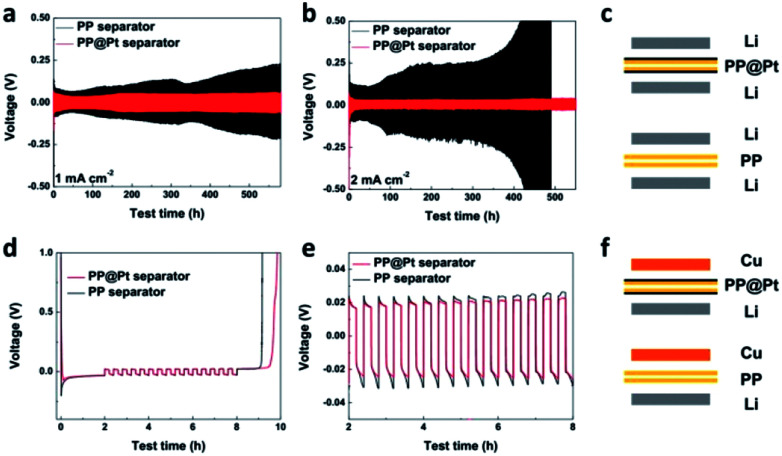
Galvanostatic cycling performance of Li/PP@Pt/Li (red) and Li/PP/Li (black) cells at a fixed current density of (a) 1 mA cm^−2^ and (b) 2 mA cm^−2^ at room temperature. (c) Schematic of the symmetric cell for the lithium plating/stripping experiment. (d) Voltage–time curves to calculate the average Coulombic efficiency of Li/Cu cells at 0.5 mA cm^−2^. (e) The enlarged view of (d) from 2–8 h. (f) Schematic of the Li/Cu cell for the Coulombic efficiency experiment. Each charge and discharge time is set as 12 min.

To study of long-time Li deposition in this system, the PP@Pt separator was evaluated by galvanostatic cycling in symmetrical Li batteries.^38^ We compared the voltage profiles and Coulombic efficiencies in Li/PP/Li and Li/PP@Pt/Li cells (Fig. 3c) at current densities of 1, 2 mA cm^−2^. Each charge and discharge time was set as 12 minutes. As shown in Fig. 3a and b, Li/PP@Pt/Li cells have stable cycling performance with the nanolayer of Pt for over 550 hours at 1 and 2 mA cm^−2^, respectively. In Li/PP/Li cells, it exhibited a gradual increase in overpotential, which reflected the large dendrites growth and micro-short-circuits.^24^ The cycling performance of symmetric Li cells at 5 mA cm^−2^ with PP@Pt separator are shown in Fig. S2,† with a stable overpotential within 13 mV for 500 hours. All Li/PP@Pt/Li cells showed the improved efficiency and suggested that nanolayer of Pt can efficiently change the mechanism of Li dendrite growth and reduce short-circuits. To investigate the Coulombic efficiency during Li plating and stripping, Li/Cu cells (Fig. 3f) were tested at a 0.5 mA cm^−2^ and finally calculated by Aurbach's method (details see in Experimental section).^39^ As shown in Fig. 3d, the initial Li loading is 1 mA h cm^−2^ (*Q*_l_), the Li loading in a deposition/stripping process is 0.1 mA h cm^−2^ (*Q*_c_). For *Q*_r_, the residual Li in the final charging process of Li/PP/Li and Li/PP@Pt/Li, is 0.575 and 0.925 mA h cm^−2^, respectively. After calculating by the equation in Experimental section, the average Coulombic efficiency of cells in PP@Pt separators was calculated to 97%, which was higher than that in the commercial PP separators (83%). To further analyze the difference of the Coulombic efficiency, we amplified the cycles of charging/discharging steps in Fig. 3e. In the PP@Pt system, Cu electrode starts to plate Li at −24 mV, and strips Li at 19 mV. However, in the commercial PP separator, the plating and stripping processes start at approximately −33 and 23 mV, respectively. It is concluded that Pt layer is the positive furtherance of the nucleation and dissolution of Li.

**Fig. 4:**
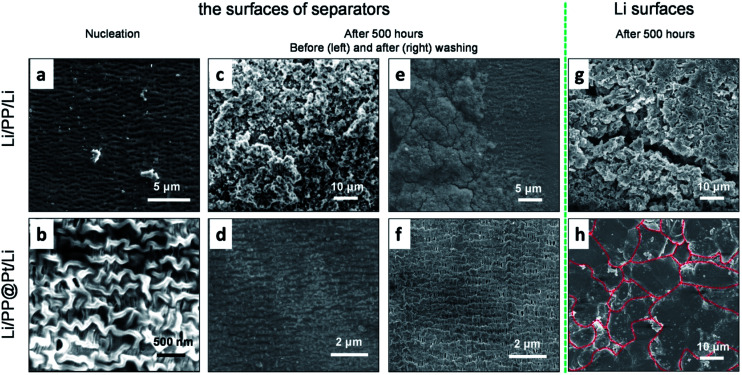
Li nuclei on the surfaces of (a) PP and (b) PP@Pt separators contacted with Li anodes side after depositing a fixed amount of Li, 0.1 mA h cm^−2^, at 1 mA cm^−2^. The morphology of the surfaces of (c) PP and (d) PP@Pt separators contacted with Li anodes side after 500 hour-cycling at 1 mA cm^−2^. (e and f) SEM images of (c) and (d) after removing Li and impurities. SEM images of Li anodes cycled for 500 hours at a current density of 1 mA cm^−2^ using (g) PP and (h) PP@Pt separators.

To further study the influence of Pt layer for the Li growth, we disassembled some of the symmetric Li cells to observe separators and the metallic Li anodes. For the sake of investigating the step of nucleation, a fixed amount of Li (0.1 mA h cm^−2^) was deposited on the objective electrode. Fig. 4a shows some large, irregular Li nuclei were inlaid in some defects of PP separator and uneven local current density can priority appear on these defects. In the PP@Pt system (Fig. 4b), homogeneous Li nuclei were distributed on the interlaced networks of PP separator with the guidance of Pt nanoparticles. Pt nanoparticles strengthened the stiffness of polymer matrix of PP, and Li nuclei improved the wettability of Li metal. Besides, the structures of nanochannels in PP separator were enhanced by the double metallic layers formed by sputtered Pt and deposited Li, ensuring efficiency transportation for Li-ions. The Li morphology of Li dendrites growth were performed on symmetric Li cells operated at a current density of 1 mA cm^−2^ after 500 hours (=1250 cycles). No Li dendrites were seen on the PP@Pt separator, while large dendrites were observed on the commercial PP separator (Fig. 4c and d). The same happened on the side of Li anodes (Fig. 4g and h), after several cycles, the Li anode using PP separator showed the appearance of large amount of Li dendrites with different shapes, while Li anode contacting with PP@Pt separator represented regularly fish-scaled flat surface. To examine the deformation degrees of separators contacting with Li dendrites, the separators above were washed by ethanol to remove Li and other impurities. In Fig. 4e, the bare PP was greatly damaged by hard Li and corroded electrolyte with loosened matrix and sparse channels. Upon the protection of Pt layer, the PP@Pt separator maintained the original structure of polymer matrix and channels (Fig. 4f). This result further verified the mechanism mentioned above.

**Fig. 5:**
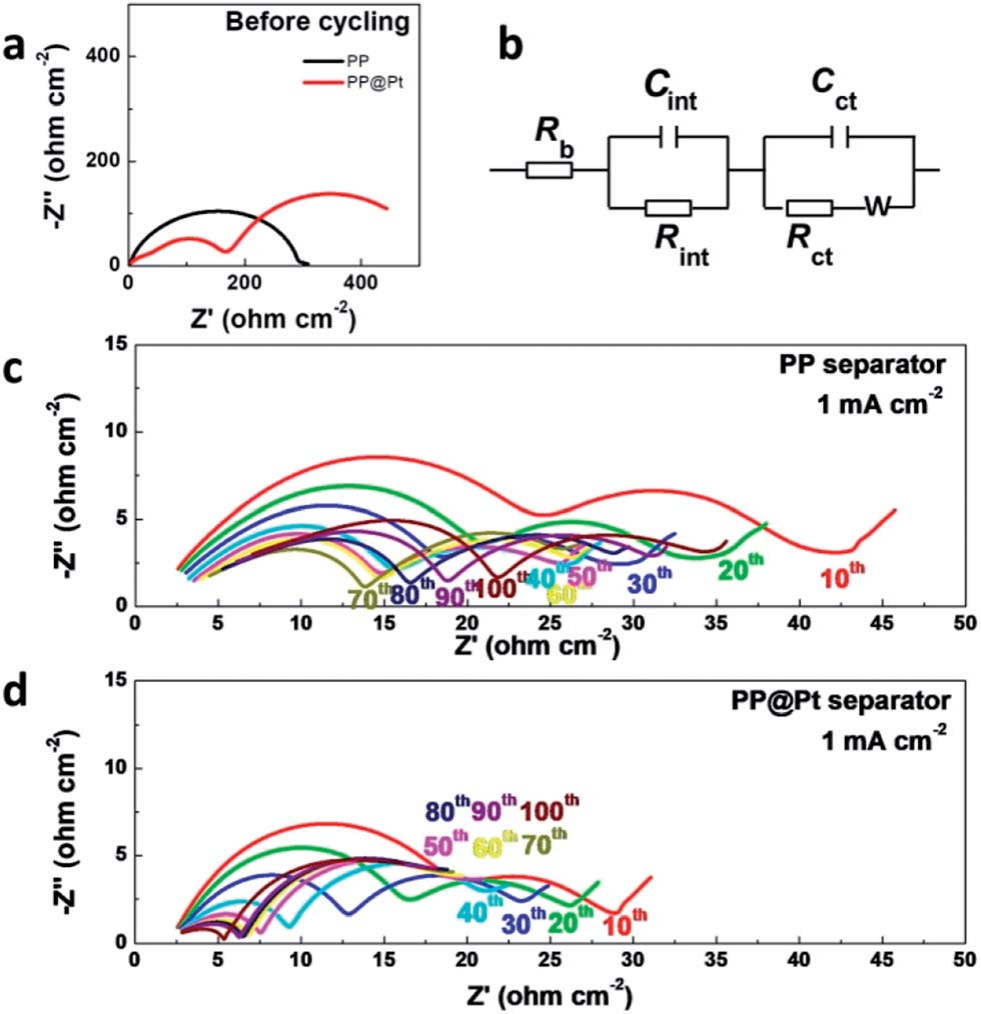
(a) EIS of Li/Li cells before cycling. (b) Equivalent circuit model for fitting impedance spectra. EIS of Li/Li cells at 0.5 mA cm^−2^ using (c) commercial PP separator, (d) PP@Pt separator.

To further prove the stability of “electrode–electrolyte” interfaces, the electrochemical impendence spectroscopy of symmetric cells using different separators were measured before and after 10, 20, 30, 40, 50, 60, 70, 80, 90 and 100 cycles at 1 mA cm^−2^. The results were fitted with equivalent circuits shown in Fig. 5b. *R*_b_, *R*_int_ and *R*_ct_ are attributed to the bulk resistor, the interfacial resistor and charge transference resistor, respectively. *C* represents a constant phase element. *W* represents a Warburg element.^40^ The *R*_b_ values of PP showed a slight increase from 1.85 to 2.92 Ω cm^−2^, which indicted the consumption of electrolyte. In conversely, the *R*_b_ values of PP@Pt is stable, with a value around 2.2 Ω cm^−2^. After 60 cycles, cells with PP@Pt showed a stable interfacial resistance around 4 Ω cm^−2^, while the resistances of PP exhibited unstable, ranging from 11 to 19 Ω cm^−2^ (Fig. 5c and d, Table S1†). Uncertain changes of interfacial resistance corresponded to the complex deformations on the interfaces.^41^ The ESI analysis demonstrated that the bidirectional growth mechanism can integrate a stable “electrode–electrolyte” interface.

**Fig. 6:**
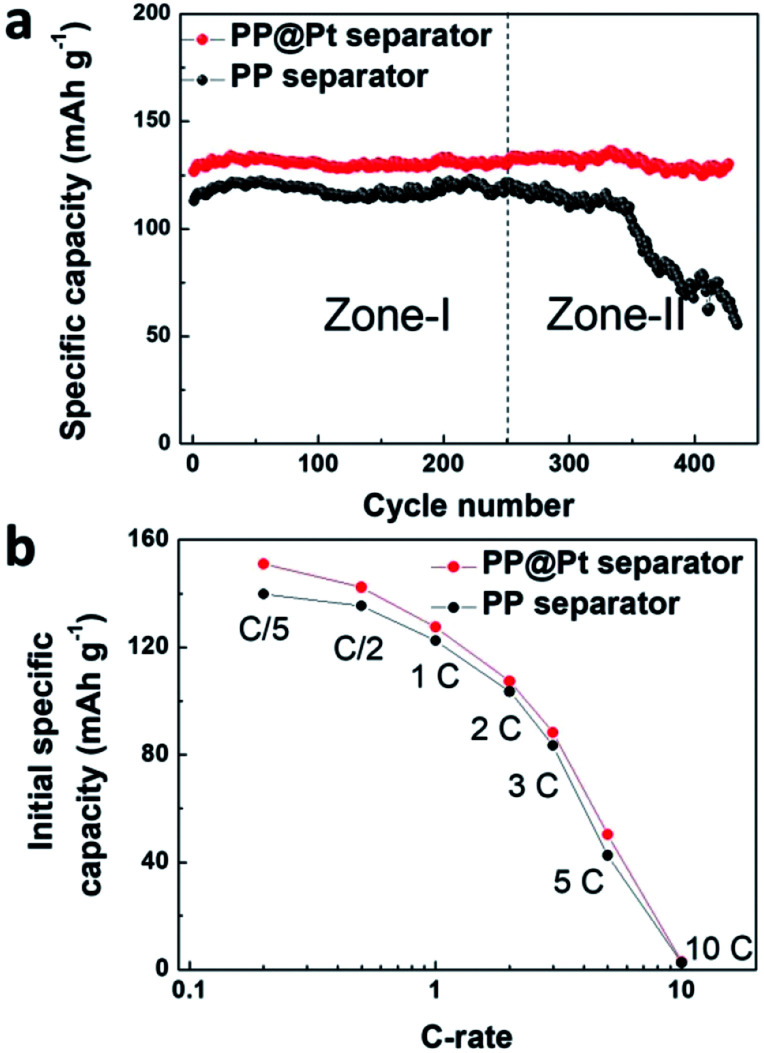
(a) Galvanostatic cycling of Li/LiFePO_4_ cells with PP@Pt separator (red) and PP separator (black) at 1C. (b) Initial capacity of Li/LiFePO_4_ cells at different C-rates with PP@Pt separator (red) and PP separator (black).

To evaluate the performance of PP@Pt separator, we performed half cells cycling of Li/LiFePO_4_ at 1C using bare PP or PP@Pt separators. As shown in Fig. 6a, the cell with commercial PP separator shows a stable cycling in Zone-I (average capacity, 118 mA h g^−1^) and a sharp decrease in Zone-II (from 121 to 73 mA h g^−1^), which is attributed mainly to the failure of Li anode and consumption of electrolyte. Conversely, the cell with PP@Pt separator can reach an average capacity of 131 mA h g^−1^ for more than 400 cycles. Fig. 6b, S3† presents the initial specific capacity at the different C-rates from 0.2C to 10C. The difference between the cells with PP and PP@Pt separators became smaller with the increase of C-rate. Cells with PP@Pt shows relatively higher capacities than cells with PP at 0.2C to 5C, which confirmed the reliability of the system in practical needs. Since the separator contains a Pt layer, the Li anode was stabilized by the bidirectional growth. Both growing Li dendrites from the two ways and some dead Li are integrated into a new stable Li anode. The design is compatible with Li metal batteries, and can facilitate the application in high-energy-density systems.

## Conclusions

In conclusion, we proposed a bidirectional growth mechanism to stable Li nucleation and deposition in Li metal battery by applying a platinum nanolayer on the side of separator contacting with Li anode. Pt nanolayers with good electronic conduction provide Li deposition sites during repeated charging/discharging. Moreover, Pt nanolayers can enhance the mechanical properties and micro-structures of commercial polypropylene separators. This system achieved the bidirectional growth of Li dendrites, which efficiently filled gaps between Pt layers and Li anodes by integrating dead Li and Li dendrites into smooth and dense Li layer. The symmetric Li/PP@Pt/Li cells exhibit low overpotentials, dense Li anode and strong tolerance under high current densities. Meanwhile, Li/LiFePO_4_ cells present excellent electrochemical performance with an average specific capacity of 131 mA h g^−1^ at 1C. The Pt modified PP separator not only ensures stable cycling performance and practical application, but also possesses ease to be fabricated, which makes it possible to be used in next-generation energy storage system, especially for advanced metallic batteries.

## Experimental

### Preparation of platinum thin film by sputtering

Pt thin film was deposited on PP separators (Celgard 2400) by an auto fine coater (JEC-3000FC). High-purity argon (Ar) was employed as carrier gas for the whole process. The PP separators were fixed on a round aluminum substrate holder that was opposite to the target. The distance between the specimen and target was 60 mm. The chamber was then evacuated until it reached a pressure under 4.0 Pa to start procedures of Pt deposition. The deposition parameters of a DC current of 30 mA and 2.5 min was applied to obtain a Pt thin film of 25 nm. After cooling the process chamber for 30 seconds, the chamber was opened and the PP@Pt separators were removed from the substrate. For preparing different Pt thicknesses, we sputtered for different durations. Thus, 12 min was estimated as 120 nm (10 × 12 nm = 120 nm) and 25 min was estimated as 250 nm (10 × 25 nm = 250 nm).

### Mechanical testing

The stress–strain tests were carried out using a Dynamic Thermomechanical Analyzer (DMA, TA Q800, USA). The preloaded force was 0.001 N and the tensile rate was 0.2 N min^−1^ and 1.5 N min^−1^ for A–A′ direction and B–B′ direction, respectively. All the samples were prepared with dimensions of 10.00 mm in length and 6.00 mm in width.

### Morphological characterizations

Scanning electron microscopy (SEM) observation was performed by using an Ultra-high Resolution Scanning Electron Microscope (SEM, Hitachi SU8020, Japan) with an acceleration voltage of 5 kV. In order to remove the effect of Pt layers, samples in this work were not sputter coated with additional Pt or C. To investigate Li surfaces after cycling, the anodes were also studied by SEM, with a special vacuum transfer box to avoid oxidation.

### Electrochemical measurements

All of the electrochemical measurements were performed by using CR2025 coin cells at room temperature. The mixture consisting of 80 wt% LiFePO_4_, 10 wt% Super P and 10 wt% PVdF were casted on aluminum foil and dried in a vacuum oven at 80 °C for 24 h. After that, the aluminum foil was punched into slices (*d* = 14 mm) with areal loading of 5.6 mg cm^−2^. The electrolyte was 1 M LiTFSI/(DME-DOL) (volume ratio = 1 : 1) (supplied by Shanghai Xiaoyuan Energy Technology Co., Ltd.). The electrolyte amount in all cells was 60 μL. The Li plating/striping tests were performed under various current densities (1, 2, 5 mA cm^−2^) by using symmetric Li cells by a Neware CT-3008 battery tester. Each charge and discharge time was set as 12 min. The Coulombic efficiency was calculated after Aurbach *et al.*^39^ by using the Li/Cu cells. The cells were initially discharged at 0.5 mA cm^−2^ for 120 min, and then charged/discharged at the same current density for 12 min/12 min for 15 cycles. In the end, the last step was a Li dissolution process interrupted when the working electrode potential exceeded 1 V *vs.* Li/Li^+^. The cycling efficiency was calculated by the following equation.*X* = [*Q*_c_ − (*XQ*_l_ − *Q*_r_)/*N*]/*Q*_c_where 100*X* is the Coulombic efficiency (%), *N* is the cycle number, *Q*_c_, *Q*_l_, *Q*_r_ are the charges involved in a single deposition/stripping process (half cycle), initial loading (massive Li deposition), and final charging (the residual Li), respectively. Electrochemical impedance spectroscopy (EIS) measurements were carried out on the Autolab (PGSTAT302N) electrochemical workstation using the Li/Li cells in the frequency range of 1.0 MHz to 0.1 Hz with an amplitude of 10 mV at room temperature for each 10 cycles within a total 100 cycles. The Li/LiFePO_4_ coin cells were measured in galvanostatic mode within a voltage range from 2.5 to 3.8 V at 1C (1.0C = 170 mA g^−1^). The C-rate capabilities were conducted at the rates of 0.2C, 0.5C, 1C, 2C, 5C, 10C and then 1C. Two pieces of separator were configured in symmetric Li cells and Li/Cu cells, while one separator was used in Li/LiFePO_4_ cells. Both PP@Pt and PP separators were used in the same way, facing the sputtered Pt surface to the surface of the Li metal or Cu electrode.

## Conflicts of interest

There are no conflicts to declare.

## Supplementary Material

RA-008-C8RA02140F-s001
