# Supplementary material for: A bidirectional growth mechanism for a stable lithium anode by a platinum nanolayer sputtered on a polypropylene separator
Source: RSC Adv. 2018 Apr 9;8(23):13034–9. doi: 10.1039/c8ra02140f (PMC9079675; doi:10.1039/c8ra02140f)
Supplement: RA-008-C8RA02140F-s001 [file RA-008-C8RA02140F-s001.pdf]

Electronic Supplementary Material (ESI) for RSC Advances.
This journal is © The Royal Society of Chemistry 2018

Electronic Supplementary Information (ESI)

A Bidirectional Growth Mechanism for Stable Lithium Anode by Platinum Nanolayer Sputtered on Polypropylene Separator

Kaihua Wen,[a,b] *Lili Liu,*[c] *Shimou Chen**[,a] *and Suojiang Zhang,**[,a]

[a]Beijing Key Laboratory of Ionic Liquids Clean Process, Key Laboratory of Green Process and Engineering, Institute of Process Engineering, Chinese Academy of Sciences, Beijing 100190, P. R. China.

[b]University of Chinese Academy of Sciences, Beijing 100049, P. R. China.

[c]Key Laboratory of Cosmetic, China National Light Industry, School of Science, Beijing Technology and Business University, Beijing 100048, China.

*Corresponding author. E-mail: chenshimou@ipe.ac.cn; sjzhang@ipe.ac.cn.

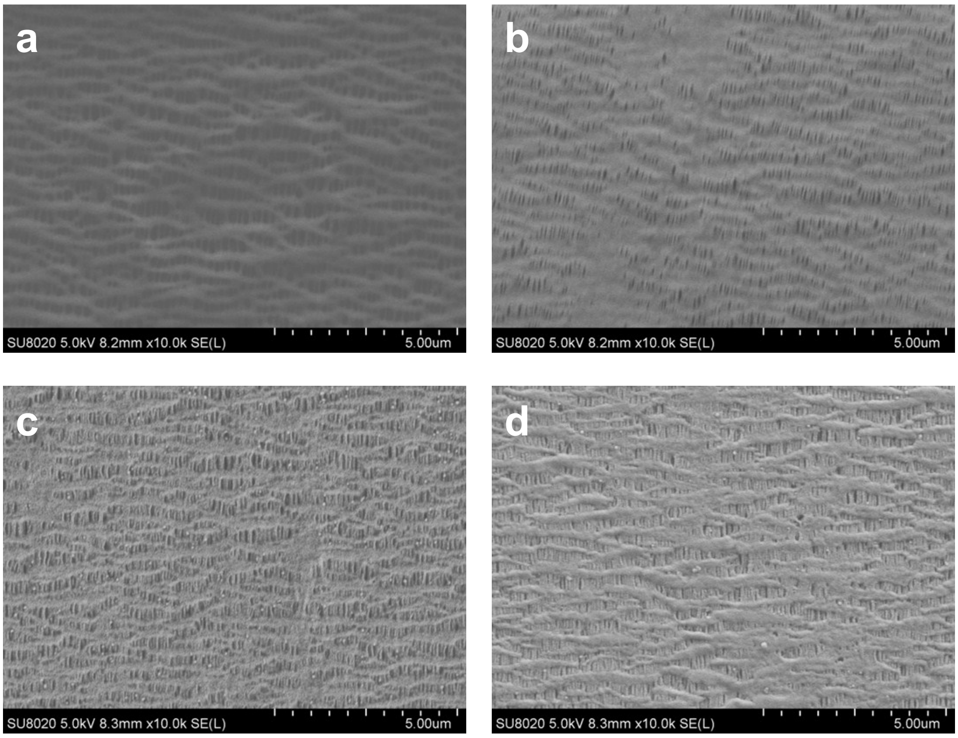

Fig. S1 SEM images of PP separators different thickness of sputtered Pt layers. (a) 0 nm. (b) 25 nm. (c) 120 nm. (d) 250 nm.

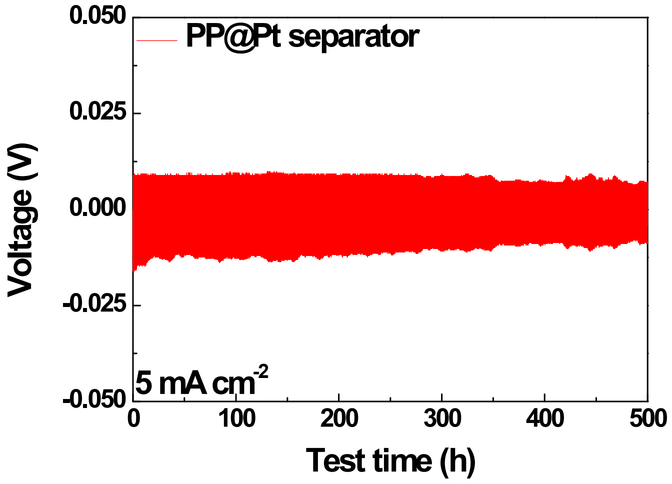

Fig. S2 Galvanostatic cycling performance of Li|PP@Pt|Li cell at 5 mA cm^{-2} at room temperature for 500 hours.

Table S1 Resistances data of Li/Li cells using PP AND PP@Pt separators obtained by fitting the spectra to the equivalent circuit presented in Fig. 5b.

Cycle number	R_b (ohm cm^{-2})		R_{int} (ohm cm^{-2})	
	PP	PP@Pt	PP	PP@Pt
0	2.62	1.87	292	167
10	1.85	2.13	22.73	17.26
20	1.98	2.25	19.02	13.96
30	2.32	2.33	13.29	10.45
40	2.45	2.20	13.42	7.01
50	2.38	2.13	12.41	5.46
60	2.65	2.33	12.21	4.59
70	2.72	2.26	11.20	3.91
80	2.45	2.18	14.03	4.26
90	2.85	2.27	16.00	4.04
100	2.92	2.15	18.89	3.28

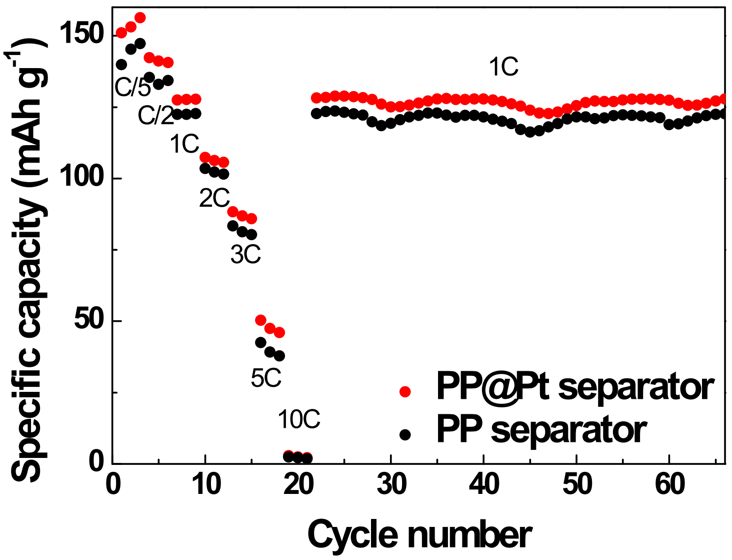

Fig. S3 Galvanostatic cycling performaxnce of Li/LiFePO_4 cells at different C-rates with PP@Pt separator (red) and PP separator (black).
